# Intracranial Cysts: An Imagery Diagnostic Challenge

**DOI:** 10.1155/2013/172154

**Published:** 2013-05-02

**Authors:** Alexandra Oprişan, Bogdan O. Popescu

**Affiliations:** Department of Neurology, Colentina Clinical Hospital, CDPC, School of Medicine, “Carol Davila” University of Medicine and Pharmacy, 19-21 Şos. Stefan cel Mare, 020125 Bucharest, Romania

## Abstract

Intracerebral cysts and cystic appearing intracerebral masses are common findings at routine cerebral imaging examination. We discuss here the most interesting aspects of some intracerebral cysts encountered in medical practice in terms of imaging, clinical and pathological description, and problems of differential diagnosis. On an almost routine basis, the neurologists have to deal with such differentials. Therefore, we aim to mention here some of the frequently encountered diagnosis problems when a patient presents with a cystic cerebral mass.

## 1. Introduction

Quite frequently, the neurologist meets a patient with a cystic brain mass revealed by imagery. This finding might be accompanied by neurological focal signs or just completely incidental information in persons investigated by CT or MRI for trivial symptoms, such as chronic headaches. However, once seen, such a structural brain abnormality needs to be diagnosed since treatment might be needed or not, and prognosis is extremely variable from one type of lesion to another. Therefore, we aim in this paper to help the clinician with the major differences in brain imagery aspects of different such cystic masses.

## 2. Metastatic Cysts

Detection of an intracerebral mass in patients with known malignant tumour strongly suggests the presence of brain metastases. In about 70% of patients with cerebral metastases, imagery demonstrates more than one lesion [1]. The most common sources of intracranial metastases in order of decreasing frequency are carcinomas of the lung, breast, malignant melanoma, carcinomas of the kidney, and carcinomas of the gastrointestinal tract [1].

The most frequent carcinomas capable to metastasize to the brain are bronchogenic carcinoma, carcinoma of the breast, choriocarcinoma, and melanoma. The latter tumour metastasizes in up to 90% of patients. Cerebral metastases occur most often at the junction of cortex and underlying white matter, roughly 80% being located in the arterial distribution zones of the cerebral hemispheres, 3% in the basal ganglia, and 15% in the cerebellum. Only 5% of the intracranial metastases are from mucinous adenocarcinomas of gastrointestinal origin, and about 50% of those are located in the posterior fossa, particularly in the cerebellum [1].

**Figure 1:**
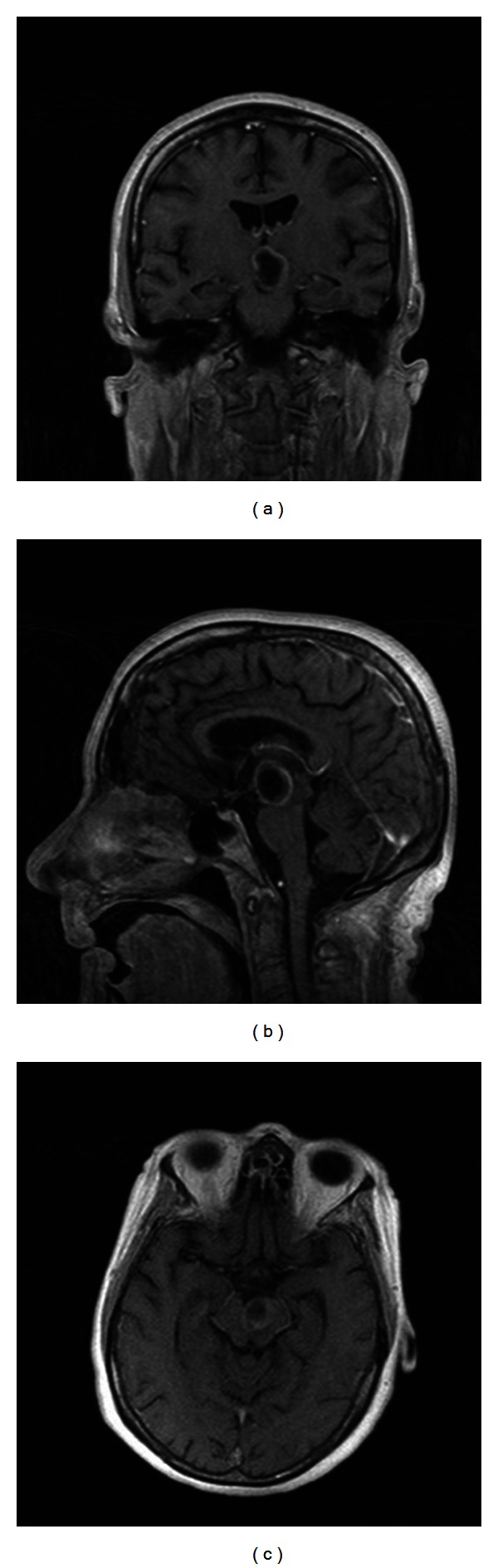
Cystic metastasis. Rim-enhancing hypointense mesencephalic lesion revealed by postcontrast T1-weighted MRI images ((a): coronal, (b): sagittal, and (c): axial slices). Department of Neurology, Colentina Clinical Hospital brain imaging archive.

**Figure 2:**
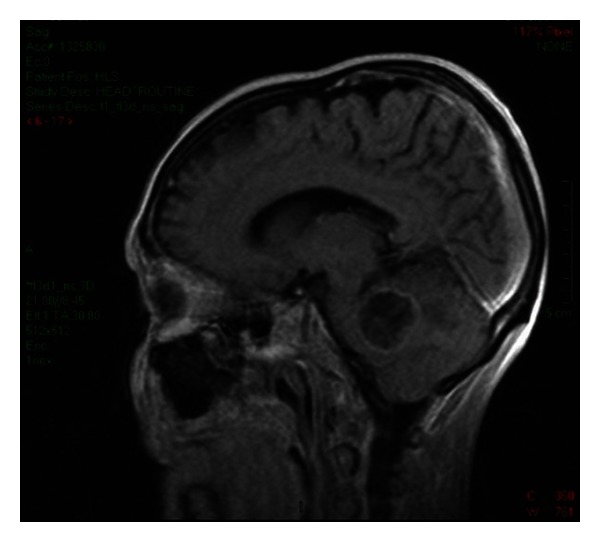
Cystic metastasis. Infratentorial, rim-enhanced hypointense lesion revealed by post-contrast T1-weighted MRI image. Department of Neurology, Colentina Clinical Hospital brain imaging archive.

On MRI examination, most intracerebral metastases show diminished signal on T1-weighted images and increased signal on T2-weighted images (see Figures 1 and 2). T1-weighted MR sequence after administration of gadolinium is the most sensitive method for evaluation of intracranial metastases, because the lesions are almost always enhanced, appearing as ring, punctuate, or solid. Some of them, especially with large size, have central necrosis, and intratumoural haemorrhage is found in about 20% of cases, more frequent in dissemination of melanomas, choriocarcinomas, carcinomas of the lung, kidney, and thyroid, in this order. Calcification is rarely found, in general being interpreted as a sign of benignity. The MRI aspects of the brain mass depend on the cellularity of the lesion, the presence of intratumoural necrosis, haemorrhage, calcification, and surrounding oedema [1].

**Figure 3:**
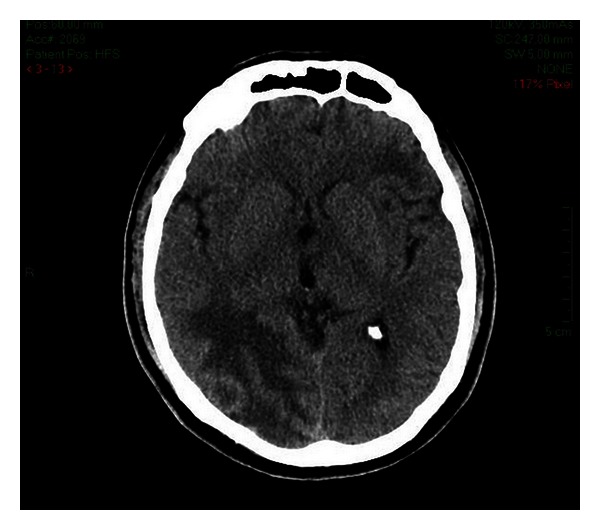
Cystic metastasis. Hypodense, well-demarcated parietal lesion with surrounding digitiform oedema revealed by native CT scan. Department of Neurology, Colentina Clinical Hospital brain imaging archive.

On CT scan without enhancement, the tumours are difficult to be detected due to their discrete appearance as rounded homogeneously isodense or, less commonly, slightly hyperdense nodules (Figure 3), surrounded by extensive vasogenic oedema, seen as hypodensity of the cerebral matter in the vicinity [1].

Contrast cerebral CT and MRI evaluation increase the specificity of diagnosis but none of them can differentiate between the lesion types. Rim-enhancing necrotic or cystic neoplasms require differential diagnosis with brain abscesses in their capsular stage, which can also occur in patients with malignancy because of the immunosuppression. Diffusion-weighted (DW) MRI and spectroscopy can be helpful in such cases. The diffusion information may be related to signal intensity or to the image map of the apparent diffusion coefficient (ADC). The ADC values obtained with DW imaging and the ADC ratio are very useful in discriminating different lesion types. In the capsular stage of brain abscesses, the CT and MRI findings are of ring-enhancing masses with surrounding oedema. A hyperintense rim of T1-weighted images corresponding to a low signal intensity rim on T2-weighted images is thought to be due to the presence of free radicals produced by activated macrophages [2]. Reduced diffusion (restricted movement of water molecules) occurs within abscesses because of highly viscous purulent fluid. The ADC measurements and DW MRI showed reduced diffusion (hyperintensity) with low ADC values in these cases [3, 4]. The ADC values may vary greatly with the age of an abscess, the course of therapy, size of the lesion, and immune status of the host [2]. A high ADC value corresponds to low signal intensity on diffusion-weighted images in cystic necrotic tumours, because the necrosis permits free diffusion of water molecules [4, 5].

However, ADC measurements have their limitations in respect to recent observations that some cystic neoplasms (primary intracerebral or metastatic) also showed low ADC values and reduced diffusion which rise another problem in differentiating them from brain abscesses. Further on, some parasitic abscesses and sterile-treated brain abscesses showed high ADC values similar to neoplasm cysts [2, 6]. The specificity of DWI for differential diagnosis between abscess and tumour is probably highest in the late capsule-formation stage or even later, because in the early stage of abscess development the findings are similar with those seen in early tumour necrosis and must be interpreted with caution [7, 8]. Using as additional criterion the T2 hypointense rim characteristics can increase the accuracy of diagnosis up to 94, 3% [2].

Magnetic resonance spectroscopy may be used for differentiation between brain tumours and abscesses. The spectral pattern of an abscess shows elevation of acetate, succinate, lactate, and amino acids such as valine, leucine, and isoleucine. These amino acids are not seen in the *in vivo* proton MR spectra of brain tumours [4, 9]. The fact that spectral changes have been observed after antibiotic treatment emphasizes the idea of performing this investigation before starting antibiotic therapy, and it also allows monitoring the disease evolution and the effectiveness of this therapy. However, MR spectroscopy has several limitations, and diffusion weight imaging is apparently a more efficient and practical method especially in small peripheral lesions, skull base lesions, and treated abscesses [4].

## 3. Cerebral Toxoplasma Abscesses

Toxoplasmosis is caused by *Toxoplasma gondii*, an intracellular parasite protozoan. In patients with AIDS toxoplasmosis is the most common opportunistic infection and the most common cause of focal brain lesions. Toxoplasmosis may generate focal brain lesions, mostly localized to the basal ganglia, but also in other brain regions. Dissemination in the spinal cord is infrequent.

The lesions can appear as (1) *necrotizing abscesses* composed of inflammatory cells, microorganisms, and areas of necrosis which extend to blood vessels and cause hemorrhage inside the mass; (2) *organizing abscesses,* with a central area of coagulative necrosis surrounded by macrophages and few microorganisms; and (3) *chronic* “*treated*” *lesions* consisting of well-demarcated nonnecrotic or cystic spaces [10].

*Imaging*. Noncontrast CT scan shows usually multiple low-density areas, and contrast CT scan can show no enhancement, nodular enhancement, or ring-like enhancement. MRI studies show hypointensity on T1-weighted images and variable intensity on T2-weighted images, due to presence of hemorrhage and/or calcification. Postcontrast T1-weighted images may exhibit a highly suggestive abscess aspect: “the target sign” with rim-enhancement and central hypointensity with a little eccentric nodule of contrast inside the mass. The lesion is usually characterized by surrounding moderate oedema in the periphery, with hypointensity on T1-weighted images and hyperintensity on T2-weighted images [11, 12]. Typical radiological findings comprise multiple, ring enhancing lesions in both cerebral hemispheres. In only approximately 15% of the cases, lesions are solitary [13]. In most of the cases, on diffusion-weighted MRI, the center of the abscess is slightly hyperintense and the wall is relatively hypointense, with hyperintense surrounding edema. Water diffusion is not restricted in the center of the toxoplasma abscess because of its necrotic tissue content [6]. At MR spectroscopy (MRS), high lipid and lactate peaks associated with a decrease in other metabolites are characteristic for toxoplasmosis [12].

*Differential Diagnosis*. The most important differential diagnosis for cerebral toxoplasmosis is primary brain lymphoma, but pyogenic abscesses and cystic metastasis can mimic it, as discussed above.

## 4. Neurocysticercosis

Neurocysticercosis is the most common form of parasitic disease involving the brain. Neurocysticercosis occurs in 60–90% of all cases of systemic cysticercosis [14]. Common locations in the brain tissue are the gray matter-white matter junction and deep sulci, and less commonly lesions can be found in subarachnoid spaces and ventricles (especially IVth ventricle). Spinal neurocysticercosis occurs infrequently, and it is almost always associated with concomitant intracranial involvement [10, 15].

The clinical presentation of the disease may be with seizures (the commonest), headache, signs of intracranial hypertension, encephalitis, chronic meningitis, cerebrovascular complications (ischemic or hemorrhagic stroke), or spinal involvement [15].

**Figure 4:**
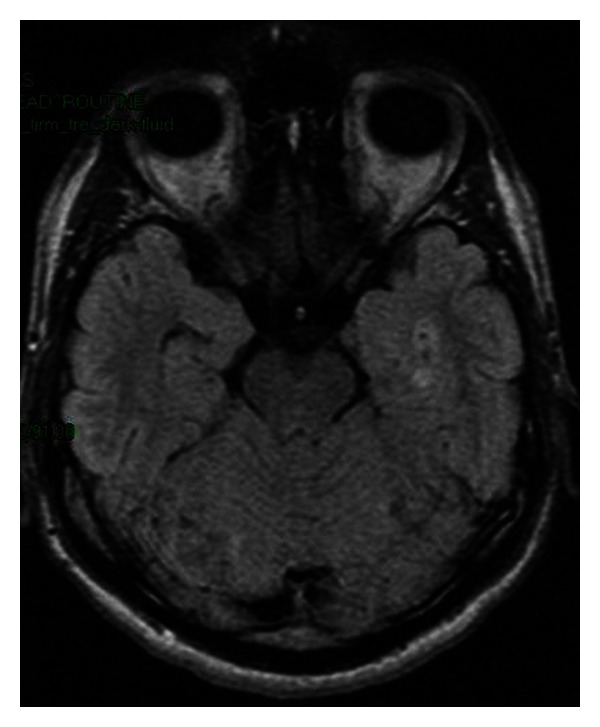
Neurocysticercosis. Round-ovalar, hyperintense, temporal lesions on FLAIR MRI sequence, suggesting “cyst with a dot” appearance. Department of Neurology, Colentina Clinical Hospital brain imaging archive.

**Figure 5:**
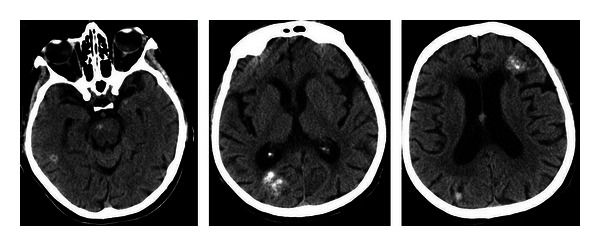
Neurocysticercosis—the nodular calcified stage. In these axial CT scan slices, multiple spontaneous hyperdense lesions are seen, some with pseudocystic aspects, with calcifications inside and without oedema or enhancement, located at the cortex/subcortical white matter border, in temporal, parietal, and frontal lobes. One pontine lesion is visible as well. Department of Neurology, Colentina Clinical Hospital brain imaging archive.

*Imaging*. The imaging findings in neurocysticercosis are variable depending on the stage of the disease. Multiple lesions in different stages of development are commonly found. In the *vesicular stage*, the cyst fluid is isodense to CSF on CT and isointense to CSF on MRI studies, with a small dot inside, because the “cyst with a dot” appearance is classically mentioned [12], as shown in Figure 4. There is no oedema and no contrast enhancement of the lesions. In the *colloidal vesicular stage*, the larva begins to disintegrate, and an intense inflammatory response takes place around the cyst, finally resulting in a fibrous capsule, which can be identified by MR imaging [15]. The cyst wall shows contrast enhancement on CT or MRI studies, and perilesional oedema occurs. Cyst fluid has increased density on CT scans and increased intensity on MRI sequences [12]. In *the granular nodular stage*, the cyst becomes a granulomatous nodule with peripheral gliosis [15]. In this stage, the lesions may show calcifications, surrounding oedema, and enhancement following contrast administration on CT scan. These lesions are identifiable as isointense on T1-weighted images and iso- to hypointense on T2-weighted images, showing nodular or ring enhancement [12]. In the *nodular calcified stage*, small calcified nodules may be seen, as shown in Figure 5. In this stage, oedema and enhancement are no longer present.

Intraventricular cysticercosis is potentially lethal due to possible acute obstructive hydrocephalus. Cysts in the basal cisterns tend to agglomerate in a racemose form, and adhesive arachnoiditis develops in this case as a local inflammatory meningeal reaction. The surrounding arteries can be invaded by inflammatory cells leading to vasculitis and cerebral infarctions or mycotic aneurysms formation [12].

*Differential Diagnosis*. The imaging differential diagnosis for neurocysticercosis includes brain abscess, primary cerebral neoplasms, cystic metastases, other parasitic infections, and tuberculosis. The differential is an important issue particularly in colloidal-vesicular stage. Identification of the scolexes in brain vesicles is pathognomonic of neurocysticercosis and essential for the right diagnosis. The pyogenic abscesses are often single lesions, whereas only about 15% of patients with neurocysticercosis have a single cyst in the nervous brain system [16]. Primary brain tumours are rarely multiple. Tuberculomas are rarely cystic and they are hypointense on T2-weighted images [14].

Intraventricular neurocysticercosis must be differentiated from choroid plexus cysts, ependymal cysts, and colloidal cysts. The latter two are discussed below in this paper.

### 4.1. Hydatid Cysts

Echinococcosis is the larval stage of *Echinococcus granulosus* infection. The disease frequently involves the liver and lung [12]. Cerebral cysts are rare, seen in only 2% of cases, and are usually solitary, spherical, and unilocular. The most common location when the infection involves CNS is the hemispheric parenchyma, particularly in the perfusion territory of the middle cerebral artery. Other rather rare possible sites are subarachnoid spaces, lateral ventricle, and cerebellum. Most cysts contain clear fluid, usually associated with small daughter cysts and a granular deposit of scolices [10].

*Imaging*. On CT or MRI examination, the hydatid lesions appear as large, spherical, cystic masses that are well demarcated from the surrounding brain parenchyma, with cyst fluid isodense with CSF on CT scans and isointense with CSF on MRI studies and lack of surrounding oedema [12]. Contrast enhancement may be seen partially or completely involving the cystic wall. The peripheral capsule of the cyst can usually be seen on MRI imaging, and calcification of the wall is better identified on CT imaging [14].

*Differential Diagnosis*. The main differential diagnosis includes other parasitic diseases involving CNS such as neurocysticercosis, where usually more numerous lesions are found, cerebral abscesses, which are surrounded by prominent oedema (as already mentioned previously), arachnoid cysts, and epidermoid cyst, with characteristic features described in the following. The identification of a single, large, unilocular cyst lesion without surrounding oedema in the parietal region of the brain is most typically suggestive for hydatid cyst [14].

### 4.2. Arachnoid Cysts

The arachnoid cyst is a benign, probably congenital lesion, localized in the intra-arachnoidal space. Usually these cysts are supratentorial. They may occur in the sylvian fissure or interhemispheric fissure and more rarely in the cisterna magna and cerebellopontine angle [17]. Occasionally they are interhemispheric under the frontal lobes or lie in the pineal region or under the cerebellum. They may have large sizes but generally do not communicate with the ventricles [18]. The arachnoid cysts account for about 1% of all intracranial masses and appear to be formed by splitting or duplication of the arachnoid membrane, active fluid secretion by the cyst wall, and slow and progressive distension caused by CSF pulsations [14]. According to the “ball valve” hypothesis, a one-way valve between arachnoid cysts and subarachnoid space might lead to its expansion, very rarely triggering a significantly serious neurological condition. Arachnoid cysts might be posttraumatic as well. Some traumatic arachnoid cysts had a latent period from head trauma to initial clinical manifestations ranging from 10 months to 6, 2 years [19]. It must also be emphasized that chronic subdural hematoma is often associated with arachnoid cysts [18] especially when the latter occur in the middle cranial fossa.

**Figure 6:**
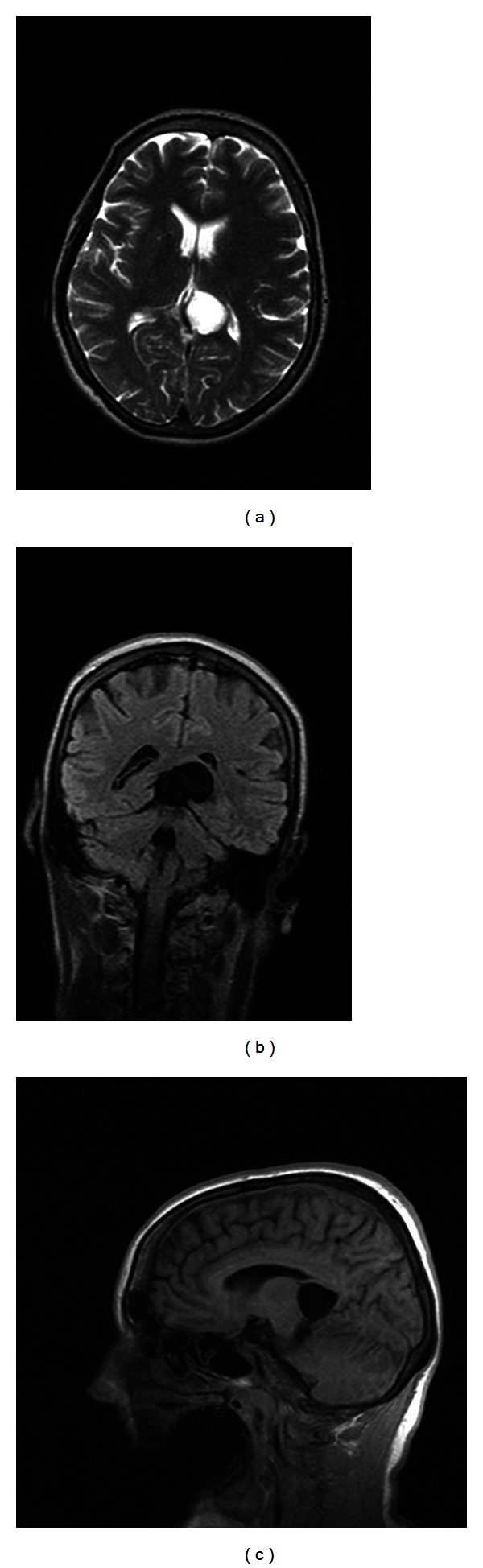
Cisterna ambiens arachnoidian cyst. (a) Round-ovalar, homogeneous, well-demarcated, hyperintense lesion with signal similar with CSF revealed by T2-weighted MRI image. (b) Round-ovalar, well-demarcated, hypointense cystic lesion with signal similar to CSF revealed by FLAIR MRI sequence. (c) Round-ovalar, homogeneous, well-demarcated, hypointense lesion with signal similar to CSF revealed by T1-weighted MRI image. Department of Neurology, Colentina Clinical Hospital brain imaging archive.

*Imaging*. The cysts are well circumscribed, having the same signal intensity as CSF at CT scans and all MRI sequences, and no contrast enhancement (see Figure 6). Occasionally, signs of hemorrhage, high protein content within the cysts occur on MRI imaging [14].

*Differential Diagnosis*. The differential diagnosis of arachnoid cysts includes epidermoid cysts, chronic subdural hematoma, and porencephalic cysts. Epidermoid cysts are hyperintense on FLAIR MRI sequence and show increased signal intensity on diffusion sequences, while arachnoid cysts have low signal intensity on both FLAIR and diffusion sequences [20]. The chronic subdural haematomas do not show the same signal intensity with CSF on MRI examination. Porencephalic cysts are CSF-filled cavities with a thin wall and surrounded by gliotic or spongiotic white matter [14].

### 4.3. Ependymal Cysts

Ependymal cysts are usually common benign cysts of the lateral ventricles. These lesions occur also in other sites, such as subarachnoid space, brainstem, juxtaventricular, spinal cord, and very rarely in the cerebellopontine angle [21, 22]. Ependymal cells line the thin walls of cysts and secrete a clear serous fluid. They have a neuroectodermal origin, and it is thought that they arise by evagination of the floor of neural tube. Ependymal cysts are usually asymptomatic, but some might become manifest with headache, seizure, or obstructive hydrocephalus [23].

*Imaging*. The lesions are isointense with CSF on MRI T1- and T2-weighted images and have nonenhancing walls [21].

*Differential Diagnosis*. The differential diagnosis includes choroid plexus cysts, but these are typically bilateral and often enhancing, arachnoid cysts, but these occur in different locations (in subarachnoid spaces) and intraventricular neurocysticercosis which show a hyperintense rim and scolexes inside the masses on FLAIR images, as discussed above [14].

### 4.4. Colloid Cysts

Colloid cysts are endodermal congenital malformations, and they represent under 1% of all intracerebral cystic masses. Most of them are found in the third ventricle at the foramen of Monroe, but they are also found in the lateral ventricle, fourth ventricle, and even outside of ventricular system. The colloid cysts are lined by a single layer of cuboidal or columnar epithelium. Occasionally mucous goblet cells are seen. The outer layer is formed by a delicate fibrous capsule. The cyst content is usually periodic acid-Schiff-positive and is composed of debris of necrotic inflammatory cells and occasional lipid droplets [17]. Colloid cysts are asymptomatic due to their small size, and they are found incidentally. However, this depends on the side of the lesions; at large sizes, they occasionally may trigger diverse neurological symptoms: neuropsychological disorder (anterior third ventricle), olfactory and gustatory hallucinations, recurrent headache, brutal neurological deterioration, and sudden death due to development of acute hydrocephalus [17].

**Figure 7:**
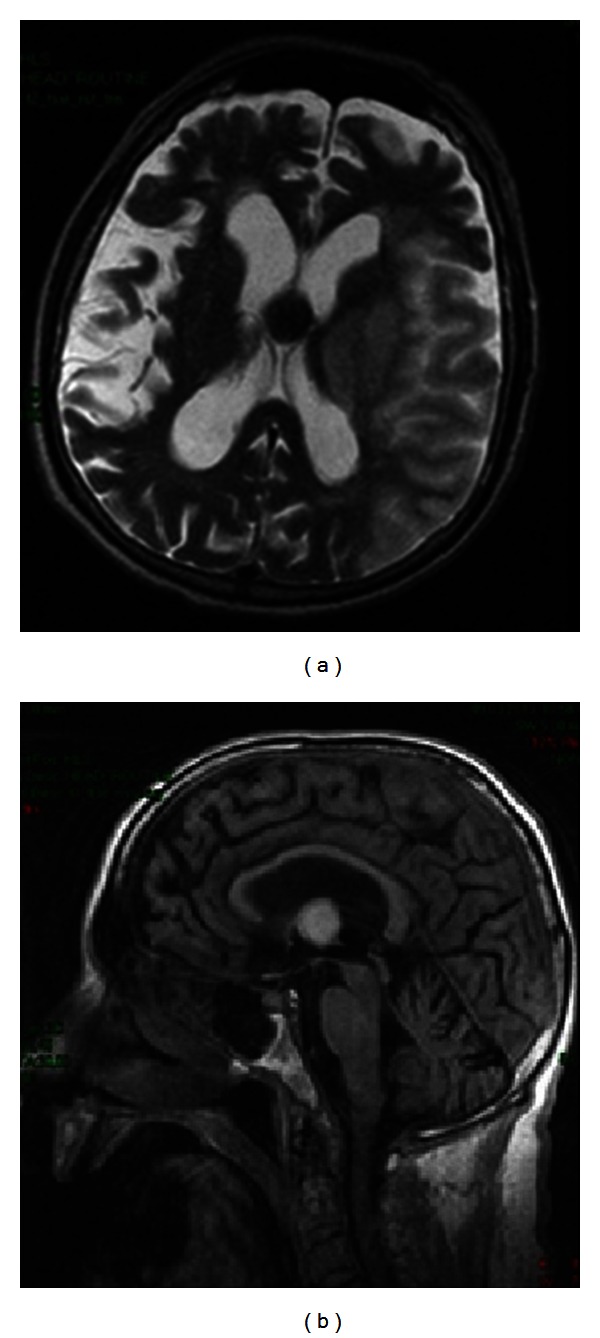
Colloid cyst. Round, well-demarcated lesion of third ventricle, hypointense on T2-weighted (a) and hyperintense on T1-weighted (b) MR images. Department of Neurology, Colentina Clinical Hospital brain imaging archive.

*Imaging*. On CT scan, the colloid cysts are usually hyperdense related to gray matter, but some of them may be hypo/isodense. After enhancement, the lesions show peripheral contrast corresponding to the capsule cyst. As shown in Figure 7, colloid cysts are hyperintense in T1-weighted images and hypointense in T2-weighted images, but they may sometimes have variable signal intensity, such as iso/hypointense in T1-weighted images. Most of them reveal increased signal intensity on FLAIR sequences and show decreased signal intensity on diffusion sequences [20].

*Differential Diagnosis*. It includes arachnoid cysts, epidermoid and dermoid cysts, and choroid plexus cysts. Colloid cysts most common location is at the foramen of Monroe, and this is very helpful in differential diagnosis. A CSF flow artifact (MR pseudocyst) may be easily mistaken for a colloid cyst. The arachnoid and ependymal cysts are isointense relative to CSF in all sequences. The choroids plexus cysts are hyperintense in T2-weighted images [20]. Epidermoid and dermoid cysts are very rarely seen in the third ventricle.

### 4.5. Epidermoid Cysts

Epidermoid cysts are benign, congenital cysts with an ectodermal origin. They represent less than 2% of primary intracranial tumors. The most common location for this cyst type is at the cerebellopontine angle (about 50%), but it also might occur in sellar and parasellar regions, diploe, rhomboid fossa, fourth ventricle/brainstem, the corpus callosum, and the pineal gland. The epidermoid cysts may develop within the frontal, parietal, or petrous bone and sometimes may destroy the inner and outer table of the cranial bone to cause soft-tissue swelling under the scalp [17].

Epidermoid cysts are well-demarcated, encapsuled lesions, with a whitish capsule of a mother-of-pearl-sheen (pearly tumor). They are lined by stratified squamous epithelium and are filled with debris, keratin, water, and cholesterol crystals. The capsule is composed of collagenous connective tissue. The cysts may compress adjacent structures or they may be firmly anchored to them, sometimes encasing vessels and nerves [17].

Most of epidermoid cysts are asymptomatic but depending on their location, they may simulate an acoustic neurinoma. They usually cause the involvement of the facial nerve followed by unilateral hearing loss. Other symptoms can also occur such as vertigo, imbalance, or trigeminal neuralgia. Occasionally, epidermoid cyst rupture may produce intense chemical meningitis. Rarely, they may be complicated by the presence of hemorrhage inside [17, 18, 24].

*Imaging*. On CT scans, epidermoid cysts appear as well-demarcated hypodense lesions that resemble CSF and do not enhance with contrast agents. Most of them show low signal on T1-weighted and high signal on T2-weighted MRI sequences. On diffusion-weighted images, they demonstrate restricted diffusion [24, 25].

*Differential Diagnosis of Epidermoid Cysts*. It includes arachnoid cyst, dermoid cyst, and cystic neoplasm. Arachnoid cysts are isointense on MRI and show no restriction on diffusion-weighted images. Dermoid cysts are filled with fat and therefore have a different appearance on MR imaging (hyperintense on T1-weighted images). Cystic neoplasms often enhance and have important vasogenic surrounding edema.

### 4.6. Dermoid Cysts

Dermoid cysts are benign, rare, congenital lesions located along the cerebrum midline and infratentorial regions, usually in the posterior cerebral fossa. These tumors have ectodermal origin as well but are rarer than epidermoid cysts, representing less than 1% of all intracranial neoplasms. Supratentorial localization is uncommon, but dermoid cysts might be identified in sellar, parasellar, temporal, and frontobasal regions. In the spinal cord, the usual affected site is the lumbosacral region where the cysts might be found extramedullary or intramedullary [17]. Dermoid cysts of the cavernous sinus are rarely reported [26]. The size of cysts is variable, and their content is sebum, desquamated epithelial cells, hair, and rarely teeth. Dermoid cysts are lined by simple stratified squamous epithelium supported by collagen. The cyst wall includes hair follicles and sebaceous and sweat glands and often shows calcifications, seen with CT scans [17]. Dermoid cysts have a slow growth rate, but they can eventually trigger clinical features such as headache, hemiparesis, visual field defects, signs of increased intracranial pressure, seizures, exophthalmos, and oculomotor palsy [26]. As for epidermoid cysts, spontaneous rupture of intracranial dermoid cysts can cause chemical meningitis [27]. Primary squamous cell carcinoma may result from malignant transformations of both dermoid and epidermoid cysts [28].

*Imaging*. Dermoid cysts appear as well-circumscribed, hypodense masses on CT scan as a result of their reach lipid content. On MRI imaging, they are hyperintense on T1-weighted sequences and give a variable signal, from hypo- to hyperintensity on T2-weighted sequences. They appear with increased signal intensity on diffusion-weighted images and FLAIR MRI sequences [20].

Serpiginous hypointense signals might be seen as well with hair-containing lesions. Enhancement has been reported but is rare, and usually there is no associated peripheral oedema [29].

*Differential Diagnosis*. It includes epidermoid cysts, craniopharyngiomas, lipomas, and teratomas. The fat content in a cystic lesion located along the midline in the posterior fossa is highly suggestive for diagnosis of a dermoid cyst. Epidermoid cysts typically resemble CSF with lack of dermal appendages. Lipomas show homogeneous fat hypodensity on CT scans and signal intensity with chemical shift artifact on MRI [14]. Brain lipomas are rare congenital lesions usually asymptomatic and with pericallosal or midline location. Craniopharyngiomas of the posterior fossa are extremely rare. In these cases, the lesion is most commonly hypointense in T1-weighted and hyperintense in T2-weighted MRI images. They may also be hyperintense in T1-weighted images due to high-protein content or hemorrhage inside, but in this case contrast enhancement is usually found [30]. On CT scans, images suggesting the presence of bone or cartilage indicate a teratoma rather than dermoid cyst. On MRI sequences, teratomas show an inhomogeneous aspect with variable signal intensity and enhancement [31].

### 4.7. Neurenteric Cysts

Neurenteric cysts are rare, benign, congenital, endodermal lesions, more commonly located in the spine than in the brain. The posterior fossa is the usual brain location, typically in the midline, anterior to the brainstem, in the cerebellopontine angle. Supratentorial localizations are very rare [32, 33]. The wall cyst has variable microscopic structural features. It is lined by single layer of pseudostratified cuboidal or columnar epithelium resembling gastrointestinal or respiratory epithelium. It may contain mucous or serous glands, smooth muscle, components of connective tissue, and lymphoid tissue. The cyst content is mainly mucoid in nature, with variable protein component [17].

Patients with neurenteric cysts may present with clinical symptoms such as headache, dizziness, cranial nerve deficits (decreased sensation in 5th nerve territory, sensorineural hearing loss, or 3rd nerve palsy), focal neurologic deficits, or seizures [32, 34].

*Imaging*. On CT scans, neurenteric cysts are iso-/hypodense lesions. Most of them are iso-/slightly hyperintense compared with CSF on T1-weighted and typically hyperintense on T2-weighted MRI sequences. The imagery appearances may be variable depending on protein content. The lesions show mild or no restricted diffusion on diffusion-weighted sequences, and rim enhancement is a very rare finding [32]. The classic neurenteric cysts are seen as well-circumscribed, nonenhancing, slightly hyperintense masses of the cerebral posterior fossa, usually located in front of the medulla.

*Differential Diagnosis*. It includes epidermoid cysts, dermoid cysts, arachnoid cysts, Rathke's cleft cysts, colloid cysts, and craniopharyngiomas. Epidermoid and dermoid cysts show moderate to intense diffusion restriction, as mentioned previously. Arachnoid cysts have the same signal intensity as CSF on all MRI sequences. Rathke and colloid cysts have a different location than neurenteric cysts. Craniopharyngiomas are hyperintense in T2-weighted MR images strongly enhancing in T1 sequence with contrast agent examination.

### 4.8. Rathke Cysts

Rathke's cysts are congenital lesions arising from remnants of the Rathke pouch. They are intra- and/or suprasellar cysts with intracystic nodules. Rathke's cysts vary in size from millimeters to 1-2 cm and are lined by a single cuboidal or columnar epithelium including cilia and goblet cells. The cyst content is mainly mucoid [14, 35]. The lesions are usually asymptomatic but eventually can trigger symptoms, due to compression of the optic chiasm, hypothalamus, or pituitary gland.

*Imaging*. Rathke's cysts might be difficult to be differentiated from other intra- or suprasellar masses on pure radiologic bases because of variable intensities of signal on MRI sequences. Still, important aspects are the lack of enhancement and absence of calcifications of the cyst. Cyst fluid shows low signal intensity to isointensity on T1-weighted and isointensity to high signal intensity on T2-weighted MR images [36]. Intracystic nodules reveal characteristic low signal intensity on T2-weighted and high signal intensity on T1-weighted MR images [37].

*Differential Diagnosis*. The most common lesions needing to be differentiated from Rathke's cysts are pituitary adenomas and craniopharyngiomas. The pituitary adenomas appear like solid, homogenous enhancing tumors. The craniopharyngiomas have mixed solid and cystic characteristics and show enhancement of the solid part [38]. More rarely, other types of masses may also occur in sellar and parasellar regions, such as epidermoid and dermoid cysts, already discussed previously. However, the intracystic nodule of Rathke's cyst visible on both T1-weighted and T2-weighted MRI sequences is very helpful in diagnosis.

## 5. Conclusions

Intracerebral cystic lesions can lead to a real diagnostic challenge for both the radiologist and the neurologist. In this respect, MRI diffusion-weighted sequences and MR spectroscopy proved to be particularly useful recently. However, these techniques are not widely available, and therefore features seen with CT scans and classical MRI sequences are still important for the differential diagnosis. In particular, sometimes it may be difficult to distinguish between cerebral abscesses and cystic necrotic tumors based on imaging. This aspect can be extremely important especially in life-threatening cases when a correct diagnosis leads to a prompt treatment. Nevertheless, in unclear cases, biopsy or surgery and subsequent pathology analysis can establish the final precise diagnosis.
